# Characterization of the APP^SAA^ mouse model for use in preclinical testing of Alzheimer's disease therapeutics

**DOI:** 10.1002/alz70855_105192

**Published:** 2025-12-24

**Authors:** Michael Sasner, Dylan Garceau, Nick Ryan, Ritesh Chidambaram, Tim Ragan, Asli Uyar, Ravi S Pandey, Amber Sanders, Sean‐Paul Gerard Williams, Gregory W Carter, Stacey J Sukoff Rizzo, Adrian L Oblak

**Affiliations:** ^1^ The Jackson Laboratory, Bar Harbor, ME, USA; ^2^ TissueVision Inc., Newton, MA, USA; ^3^ University of Pittsburgh School of Medicine, Pittsburgh, PA, USA; ^4^ Stark Neurosciences Research Institute, Indiana University School of Medicine, Indianapolis, IN, USA

## Abstract

**Background:**

The APP^SAA^ mouse was developed to provide a model of Alzheimer's disease (AD) that is accessible for preclinical research without licensing restrictions, or artifacts due to transgenic overexpression (Xia et al, 2022). We have now characterized numerous clinically relevant phenotypes throughout aging to establish the APP^SAA^ mouse as a useful model for translational research, and to enable appropriate selection of stage of disease and experimental readouts for preclinical testing programs.

**Method:**

Aβ plaques were labeled in vivo with Methoxy‐X04 and brains were processed on the TissueCyte Serial Two‐Photon Plus (STP+) imaging platform. Indexed brain sections were then used for iterative immunolabeling (IBEX; see Radtke et al, Nat Protocols 2022) to detect neuroinflammatory and vascular responses to amyloid on representative sections and remapped to the original 3D whole‐brain dataset. Brain homogenates were analyzed by transcriptomics and plasma was evaluated for biomarkers, metabolomics and lipidomics. Brain spatial transcriptomics at 9 months of age was performed using 10X Genomics Xenium platform. Cognition was assays using touch screen chambers.

**Result:**

Amyloid plaques are first detected in cortical regions at 4 months of age and accumulate up to about 12 months. Plaques are parenchymal at early stages, with vascular amyloid detected after about 15 months of age. Bulk brain transcriptomics exhibits age‐dependent correlations to AMP‐AD differentially regulated pathways, with strongest correlation to immune modules. Gene expression signatures and protein markers associated with microglia‐ and astrocyte‐driven neuroinflammatory responses were localized to regions near plaques. Handling‐induced seizures were observed during daily cognitive training in subjects after 12 months of age but not at younger ages. By 12 months of age, we observed mild learning impairments in the 5‐choice serial reaction time task as well as dendritic spine loss.

**Conclusion:**

The APP^SAA^ model is a more translationally relevant model than transgenic overexpression models for studying early onset amyloidosis and microglial activation, and is available without legal restrictions. Here we demonstrate key assays and appropriate ages/disease stages for preclinical testing.

